# Diphtheretic Paralysis

**Published:** 1890-02-22

**Authors:** 


### THE PRACTITIONER'S OUTLOOK AND RETROSPECT.

[*The Editor will be glad to receive offers of co-operation and contributions from members of the profession. There is no doubt that much valuable material full of interest and instruction is at present lost to science. This is largely so in the case of* (1) *the younger and less-known members of the hospital staff* (2) *those connected with cottage hospitals, and* (3) *the great body of medical practitioners not attached to any institution. The co-operation of all is cordially invited to enable the Editor to make* THE HOSPITAL *of the utmost possible use and value to the profession. Specialists willing to review books on their special subjects are invited to communicate this fact at once. All letters should be addressed to* THE EDITOR, THE LODGE, PORCHESTER SQUARE, LONDON, W.]

### MEDICINE.

### DIPHTHERITIC PARALYSIS.

Dr. Foord Caigen reports two rapidly fatal cases of diphtheretic paralysis. The first case was that of a gardener, æt. 25, who on May 22nd suffered from ordinary pharyngeal diphtheria. On July 1st he again felt unwell, having difficulty of swallowing, numbness in the fingers and feet, hoarse voice, muscular power of arms weak, patella reflex exaggerated. The case terminated by sweating, cyanosis, dilated pupils, and coma. Twenty-four hours before his death the man was at work. The second case occurred in a child, æt. five, admitted August 28th with faucial diphtheria. The case was a severe one. On September 17th the patellar reflex disappeared. On October 2nd food returned through the nose, and the palate was immovable. On the 14th there was double ptosis. On the 15th, inability to swallow, voice indistinct, and dyspnœa; towards evening cyanosis. Death occurred that night. It is remarkable that in both cases the symptoms were serious only shortly before death.

